# Living alone decreased calcaneus ultrasound *T*-score in a large Taiwanese population follow-up study

**DOI:** 10.3389/fpubh.2022.1004794

**Published:** 2022-10-05

**Authors:** Ting-Yi Lin, Szu-Chia Chen, Jiun-Hung Geng, Hui-Ju Tsai

**Affiliations:** ^1^Department of Post Baccalaureate Medicine, Kaohsiung Medical University, Kaohsiung, Taiwan; ^2^Department of Internal Medicine, Kaohsiung Municipal Siaogang Hospital, Kaohsiung Medical University, Kaohsiung, Taiwan; ^3^Division of Nephrology, Department of Internal Medicine, Kaohsiung Medical University Hospital, Kaohsiung Medical University, Kaohsiung, Taiwan; ^4^Faculty of Medicine, College of Medicine, Kaohsiung Medical University, Kaohsiung, Taiwan; ^5^Research Center for Precision Environmental Medicine, Kaohsiung Medical University, Kaohsiung, Taiwan; ^6^Department of Urology, Kaohsiung Municipal Siaogang Hospital, Kaohsiung Medical University, Kaohsiung, Taiwan; ^7^Department of Urology, Kaohsiung Medical University Hospital, Kaohsiung Medical University, Kaohsiung, Taiwan; ^8^Department of Family Medicine, Kaohsiung Municipal Ta-Tung Hospital, Kaohsiung Medical University, Kaohsiung, Taiwan; ^9^Department of Family Medicine, Kaohsiung Medical University Hospital, Kaohsiung Medical University, Kaohsiung, Taiwan; ^10^Community Health Development Center, Kaohsiung Municipal Ta-Tung Hospital, Kaohsiung Medical University, Kaohsiung, Taiwan; ^11^Department of Family Medicine, School of Medicine, College of Medicine, Kaohsiung Medical University, Kaohsiung, Taiwan

**Keywords:** living alone, *T*-score, osteoporosis, Taiwan Biobank, calcaneus ultrasound

## Abstract

**Background:**

Osteoporosis is associated with many serious health conditions that have a severely negative impact on quality of life, as well as higher rates of morbidity and mortality. Due to the aging society and low birth rate in Taiwan, an increasing number of people are living alone. This longitudinal study was aimed to assess the relationship between living alone and calcaneus ultrasound *T*-score in a large cohort in Taiwan.

**Methods:**

A total of 118,853 participants enrolled in the Taiwan Biobank since 2008 to 2016, who had complete calcaneus ultrasound examinations were collected in the baseline study. Of these participants, 26,850 received complete follow-up measurements after a median of 4 years. The *T*-score (g/cm^2^) of the calcaneus in the non-dominant foot was measured using ultrasound. Changes in the calcaneus ultrasound *T*-score (Δ*T*-score) were calculated as follow-up *T*-score minus baseline *T*-score. We analyzed these data in 2022. We used multivariable linear regression analysis to investigate correlation between living alone with baseline *T*-score and Δ*T*-score. We also carried out separate analyses for men and women.

**Results:**

The mean age of the participants was 49.89 ± 10.95 years, and multivariable analysis showed that living alone was significantly correlated to low baseline *T*-score in whole cohort (β = −0.040; *p* = 0.012) and women (β = −0.055; *p* = 0.023). Furthermore, living alone (coefficient β = −0.049; *p* = 0.048) was significantly correlated to a low Δ*T*-score after 4 years of follow-up.

**Conclusion:**

In this large population-based longitudinal study, living alone may be related to low baseline calcaneus ultrasound *T*-score and Δ*T*-score. Adopting long-term community-based care policies to increase the activity of people living alone may help to prevent osteoporosis and decrease the risk of fractures in Taiwan.

## Introduction

Osteoporosis is a major public health issue, and its prevalence is increasing worldwide (1). It is a skeletal disorder characterized by compromised bone strength and an increased risk of fractures, in particular of the spine and hip, with around nine million osteoporosis-associated fractures reported annually (2). Hip and vertebral fractures are a major cause of morbidity, mortality, and socioeconomic burden (3). They are particular debilitating in the elderly, and can result in loss of confidence, independence, and decreased quality of life, as well as high hospitalization and rehabilitation expenditure (3, 4). The incidence of osteoporosis and associated fractures increases with age, and the lifetime risk of an osteoporosis-related fracture in those aged 50 years is very high, at 13%−22% in men and 40%−50% in women (5).

Risk factors for osteoporosis and osteoporotic fractures contain age, sex, race, early menopause, family history of fractures, clinical disease, lifestyle, and medication (1, 4, 6–9). A lack of exercise, poor nutritional status such as calcium and vitamin D deficiency, smoking, and excessive alcohol consumption are common lifestyle factors associated with osteoporosis (1, 6). The clinical risk factors include diabetes mellitus (DM), chronic obstructive pulmonary disease, chronic kidney disease, rheumatoid arthritis, chronic liver disease, and obstructive sleep apnea (4, 10–14). Living status and social integration have also been reported to be important factors associated with an older person's health (15). However, few studies have explored the relationship between environmental factors and osteoporosis.

The incidence of osteoporosis has increased rapidly in Taiwan along with the aging population. In addition, the aging society and low birth rate have resulted in an increase in the number of people living alone. According to Taiwan National Development Council, Taiwan has been an aged society since 2018, and it is estimated to be a “super-aged” society by 2025 (16). The Taiwan Ministry of the Interior Real Estate Information Platform reported that a total of 498,697 older adults lived alone in 2021, which is 91.22% higher than 10 years previously (17). Elderly individuals living alone have been related to poor health-related quality of life, depression, smoking, drinking, poor nutritional status, and worse prognosis of systemic diseases, all of which may increase the risk of osteoporosis (18). For example, a 3-year cohort study of 288 older Chinese adults residing in suburban areas found that living alone was associated with a high risk of osteoporosis (19). In addition, a cross-sectional Korean study reported that individuals living alone in rural areas had a significantly lower lumbar spine bone mineral density (BMD) (18). Conversely, another cross-sectional research in China showed that elderly individuals living alone had better functional status, which may have decreased the risk of osteoporosis (20). Consequently, the association between living alone and osteoporosis is still inconsistent, and no studies have been conducted in Taiwan to explore the relationship between living status and the risk of osteoporosis. Therefore, in this study we identified the association between living alone and calcaneus ultrasound *T*-score using data from the Taiwan Biobank (TWB). Furthermore, we used longitudinal data to evaluate the link between living alone and changes in calcaneus ultrasound *T*-score.

## Methods

### Study population

The participants in this study were collected from the TWB, a population-based research database established in 2008 (21). Inclusion criteria for TWB was comprised of adults aged 30 to 70 years who had willingness to receive complete examination. Subjects with prior cancer history were excluded (21). Details of the TWB have been described in previous studies (22). In brief, the goal of the TWB is to promote strategies to prevent various disorders, ameliorate treatment therapies, and assist in the analyses of specific genes and biomarkers. All participants in the TWB sign informed consent forms, and then provide information on diet, personal and family medical histories, and lifestyle factors (currently smoking and exercise habit) during face-to-face interviews *via* a standardized questionnaire. Participants are also asked to undergo a physical examination and provide a blood sample. Details on the TWB are available on its official website (https://taiwanview.twbiobank.org.tw/index).

**Figure 1:**
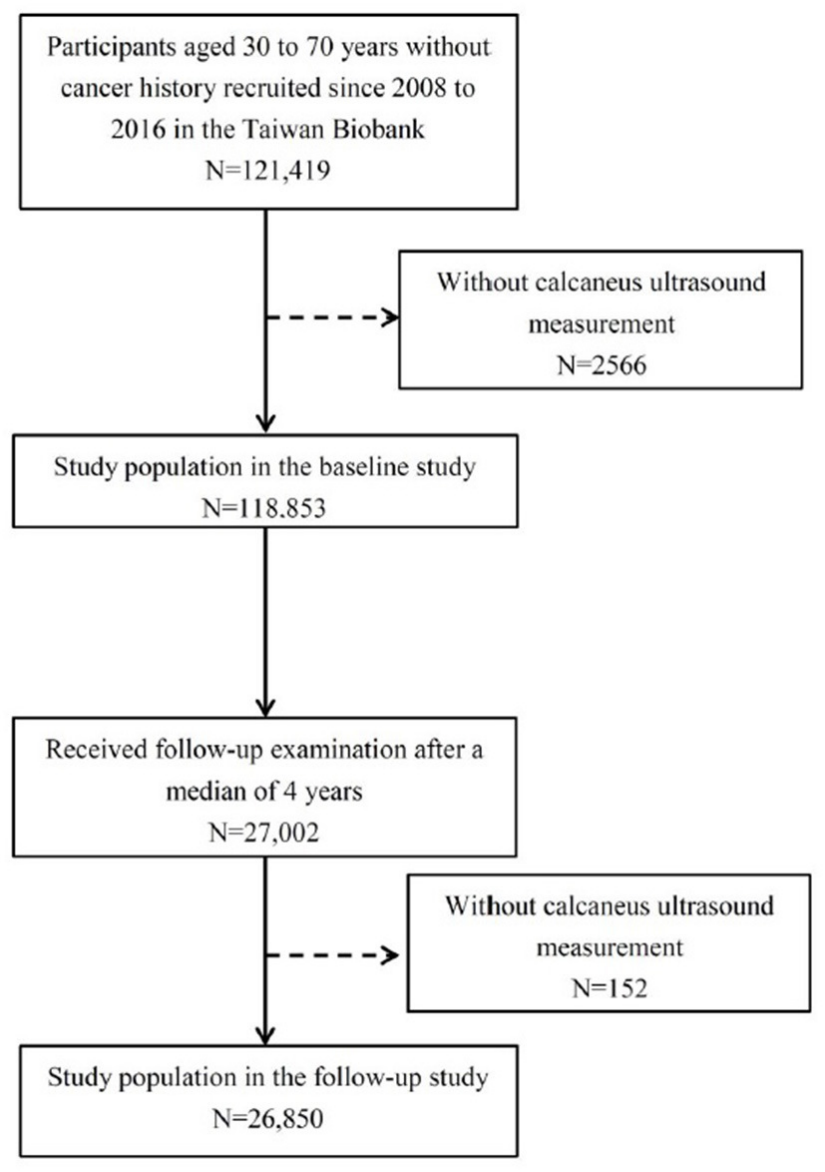
Flow chart of study population.

In this study, we screened 121,419 TWB participants during the period between 2008 and 2016, of whom 2,566 had incomplete ultrasound data and were excluded. Subsequently, we included 118,853 participants with complete ultrasound measurements into the baseline study. Of these participants, 27,002 were followed for a median of 4 years. After excluding a further 152 participants who did not have complete follow-up ultrasound measurements, the 26,850 were enrolled in the follow-up study (Figure 1). These data was analyzed in 2022.

### Collection of anthropometric, demographic, medical and laboratory variables

Demographic and medical variables of the participants including age, sex, living status, current smoking status, exercise habit, DM, and hypertension were recorded during the period between 2008 and 2016. Anthropometric [body height, weight and body mass index (BMI, kg/m^2^)] and laboratory [overnight fasting serum sugar, total cholesterol, high-density lipoprotein cholesterol (HDL-C), low-density lipoprotein cholesterol (LDL-C), triglycerides (TG), serum creatinine and uric acid (UA)] variables were also collected. Estimated glomerular filtration rate (eGFR) was calculated using the four-variable modification of diet in renal disease study equation: eGFR (ml/min/1.73 m^2^) = 175 × (serum creatinine) – 1.154 × (age) – 0.203 × (0.742 if female) × (1.212 if African American) (23).

In addition, the demographic and medical variables of men and women were separately analyzed. The age of menopause, the number of postmenopausal women, production history, the number of nursing children and breast-feeding experience were included in the table of characteristics of women.

### Assessment of calcaneus ultrasound *T*-score

Ultrasound measurements of the calcaneus were performed in the non-dominant foot using an Achilles InSight ultrasound system (GE, Madison, WI), and *T*-scores (g/cm^2^) were calculated as (the participant's mean *T*-score minus the mean *T*-score of a normal young-adult population) divided by the standard deviation of a normal young-adult population (24). Changes in *T*-scores (Δ*T*-score) were calculated as: follow-up *T*-score minus baseline *T*-score.

### Ethics statement

Ethical approval for the TWB was granted by the Institutional Review Board (IRB) on Biomedical Science Research, Academia Sinica, Taiwan, and the Ethics and Governance Council of the TWB. This research was approved by the IRB of Kaohsiung Medical University Hospital (KMUHIRB-E(I)-20210058).

### Statistical analysis

Data are shown as number (percentage) or mean (±standard deviation). Differences in categorical variables were analyzed using the chi-square test, and differences in continuous variables were analyzed using the independent *t*-test. One-way analysis of variance was used for multiple comparisons. Multivariable linear regression model was used to investigate relationships between living status with baseline *T*-score and Δ*T*-score. Because men and women may have different influencing factors on osteoporosis, we carried out separate analyses for men and women. *p*-values < 0.05 were considered to be statistically significant. The statistical analyses were performed with SPSS version 26.0 (SPSS Inc. Chicago, IL, USA).

## Results

The mean age of the 118,853 participants (42,656 males and 76,197 females) was 49.89 ± 10.95 years. The participants were divided into two groups according to living status, namely living alone (*n* = 9,572, 8.05%) and living with others (*n* = 109,281, 91.95%).

### Comparisons of clinical characteristics according to living status

Comparisons of the clinical characteristics between the two living status groups are shown in Table 1. Compared to the living with others group, the living alone group were predominantly female, had a lower BMI, higher rate of DM, higher fasting glucose and total cholesterol levels, lower HDL-C and UA levels, and lower eGFR.

**Table 1:** Comparison of clinical characteristics among participants according to living condition.

**Characteristics**	**Living alone *N* = 9,572**	**Living with others *N* = 109,081**	***p*-Value**
Age (year)	49.98 ± 11.79	49.88 ± 10.87	0.366
Female (%)	69.5	63.6	<0.001
Male (%)	30.5	36.4	<0.001
BMI (kg/m^2^)	24.01 ± 3.97	24.24 ± 3.77	<0.001
Smoking history (%)	28.1	27.2	0.060
Exercise habit (%)	41.5	40.5	0.061
Hypertension (%)	12.0	12.3	0.381
Diabetes mellitus (%)	5.7	5.1	0.006
Fasting glucose (mg/dl)	96.58 ± 25.12	95.83 ± 20.23	0.001
Total cholesterol (mg/dl)	196.91 ± 37.29	195.58 ± 35.69	<0.001
HDL-cholesterol (mg/dl)	54.47 ± 13.41	55.57 ± 13.86	<0.001
LDL-cholesterol (mg/dl)	120.88 ± 32.70	120.97 ± 31.67	0.791
Triglyceride (mg/dl)	116.75 ± 113.84	115.39 ± 92.13	0.175
Uric acid (mg/dl)	5.35 ± 1.40	5.43 ± 1.43	<0.001
eGFR (ml/min/1.73 m^2^)	102.81 ± 23.88	103.36 ± 23.88	0.033
Baseline *T*-score	−0.41 ± 1.69	−0.39 ± 1.62	0.234
Δ*T*-score	−0.33 ± 1.03	−0.26 ± 1.00	0.004

BMI, body mass index; HDL, high-density lipoprotein; LDL, low-density lipoprotein; eGFR, estimated glomerular filtration rate.

### Comparisons of clinical characteristics between men and women

Comparisons of the clinical characteristics between men and women are shown in Table 2. The mean age of the 42,656 men and 76,197 women was 49.89 ± 11.37 and 49.89 ± 10.71 years respectively. There were 49.3% postmenopausal women and the mean age of menopause was 49.4 ± 5.0 years. Compared to men, women had higher prevalence of living alone, lower BMI, lower rate of hypertension and DM, less exercise habit, less cigarette use, lower fasting sugar, LDL-C, TG, and UA levels, and higher total cholesterol, HDL-C, eGFR and baseline *T*-score (*p*-value < 0.001; Table 2).

**Table 2:** Comparison of clinical characteristics among men and women.

**Characteristics**	**Woman *N* = 76,197**	**Man *N* = 42,656**	***p*-Value**
Age (year)	49.89 ± 10.71	49.89 ± 11.37	0.981
Living alone (*N*, %)	6,654 (8.7)	2,918 (6.8)	<0.001
BMI (kg/m^2^)	23.57 ± 3.76	25.37 ± 3.55	<0.001
Smoking history (%)	10.3	57.4	<0.001
Excercise habit (%)	39.6	42.4	<0.001
Hypertension (%)	9.7	16.8	<0.001
Diabetes mellitus (%)	4.2	6.8	<0.001
Fasting glucose (mg/dl)	93.98 ± 18.78	99.31 ± 23.29	<0.001
Total cholesterol (mg/dl)	197.79 ± 36.06	191.92 ± 35.09	<0.001
HDL-cholesterol (mg/dl)	58.24 ± 13.24	47.99 ± 11.11	<0.001
LDL-cholesterol (mg/dl)	120.51 ± 31.90	121.78 ± 31.47	<0.001
Triglyceride (mg/dl)	103.05 ± 74.50	137.74 ± 118.19	<0.001
Uric acid (mg/dl)	4.86 ± 1.12	6.42 ± 1.36	<0.001
eGFR (ml/min/1.73 m^2^)	108.65 ± 24.46	93.78 ± 19.46	<0.001
Baseline *T*-score	−0.23 ± 1.69	−0.66 ± 1.46	<0.001
Δ*T*-score	−0.32 ± 1.06	−0.16 ± 0.89	<0.001
Postmenopausal women (%)	49.3	–	–
Age of menopause	49.4 ± 5.0	–	–
Production history (%)	96.3	–	–
Number of nursing children	2.3 ± 0.8	–	–
Breast-feeding experience (%)	69.8	–	–

BMI, body mass index; HDL, high-density lipoprotein; LDL, low-density lipoprotein; eGFR, estimated glomerular filtration rate.

### Correlation between living status and baseline *T*-score in the whole study cohort

The correlation between living status with baseline calcaneus ultrasound *T*-score in the whole study cohort (*n* = 118,853) were analyzed using multivariable linear regression analysis (Table 3). After adjusting for living status, age, sex, smoking status, exercise habit, DM, hypertension, BMI, fasting glucose, triglycerides, total cholesterol, eGFR, and UA, living alone [β = −0.040; 95% confidence interval (CI) = −0.071 to −0.009; *p* = 0.012] was significantly associated with a low baseline *T*-score. In addition, age (β = −0.061; *p* < 0.001), male sex (β = −0.526; *p* < 0.001), smoking (β = −0.024; *p* = 0.033), a history of hypertension (β = −0.067; *p* < 0.001), cholesterol (β = −0.001; *p* < 0.001), TG (β = −0.001; *p* < 0.001), UA (β = −0.032; *p* < 0.001) and eGFR (β = −0.005; *p* < 0.001), were negatively associated with baseline *T*-score. A history of DM (β = 0.092; *p* < 0.001), exercise habit (β = 0.242; *p* < 0.001) and BMI (β = 0.053; *p* < 0.001) were positively related to baseline *T*-score.

**Table 3:** Association of living condition with baseline calcaneus ultrasound ***T*-**score in all study participants (*n* = 118,853) using multivariable linear regression analysis.

**Variables**	**Total**		**Woman**		**Man**	
	**Multivariable**					
	**Unstandardized coefficient (95% CI)**	***p*-Value**	**Unstandardized coefficient (95% CI)**	***p*-Value**	**Unstandardized coefficient (95% CI)**	***p*-Value**
Living alone	−0.040 (−0.071, −0.009)	0.012	−0.055 (−0.103, −0.008)	0.023	0.008 (−0.044, 0.061)	0.753
Age (per 1 year)	−0.061 (−0.061, −0.060)	<0.001	−0.048 (−0.050, −0.046)	<0.001	−0.038 (−0.039, −0.036)	<0.001
Male *vs*. female	−0.526 (−0.550, −0.503)	<0.001	–	–		–
Smoking history	−0.024 (−0.046, −0.002)	0.033	−0.007 (−0.046, 0.031)	0.711	−0.159 (−0.186, −0.131)	<0.001
Exercise habit	0.242 (0.224, 0.261)	<0.001	0.209 (−0.184, 0.234)	<0.001	0.329 (0.301, 0.358)	<0.001
Diabetes mellitus	0.092 (0.049, 0.136)	<0.001	0.124 (0.062, 0.186)	<0.001	0.057 (−0.004, 0.118)	0.069
Hypertension	−0.067 (−0.095, −0.039)	<0.001	−0.044 (−0.084, −0.005)	0.028	−0.134 (−0.173, −0.096)	<0.001
BMI (per 1 kg/m^2^)	0.053 (0.050–0.055)	<0.001	0.057 (0.054, 0.061)	<0.001	0.032 (0.028, 0.036)	<0.001
**Laboratory parameters**
Fasting glucose (per 1 mg/dl)	0 (−0.001, 0)	0.073	0 (−0.001, 0)	0.260	−0.00003 (−0.001, 0.001)	0.917
Total cholesterol (per 1 mg/dl)	−0.001 (−0.001, 0)	<0.001	0 (0, 0.001)	0.018	0 (0, 0.001)	0.302
Triglyceride (per 1 mg/dl)	−0.001 (−0.001,−0.001)	<0.001	0 (−0.001, 0)	<0.001	0 (−0.001, 0)	<0.001
Uric acid (per 1 mg/dl)	−0.032 (−0.039,−0.024)	<0.001	0.007 (−0.004, 0.019)	0.220	−0.012 (−0.023, −0.002)	0.025
eGFR (per 1 ml/min/1.73 m^2^)	−0.005 (−0.006,−0.005)	<0.001	−0.005 (−0.005, −0.004)	<0.001	−0.006 (−0.007, −0.005)	<0.001
Menopause	–	–	−0.747 (−0.708, −0.786)	<0.001	–	–
Production history			−0.107 (−0.171, −0.043)	0.001	–	–
Breast-feeding experience			0.001 (−0.025, 0.027)	0.960	–	–

BMI, body mass index; eGFR, estimated glomerular filtration rate.

### Correlation between living status and baseline *T*-score in the men and women

We separately analyzed the correlation between liver status with baseline calcaneus ultrasound *T*-score in men and women (Table 3). For women, after adjusting for living status, age, smoking status, exercise habit, DM, hypertension, menopause, production history, breast-feeding experience, BMI, fasting glucose, triglycerides, total cholesterol, eGFR, and UA, living alone (β = −0.055; 95%CI = −0.103 to −0.008; *p* = 0.023) was significantly associated with a low baseline *T*-score. This result was consistent with the whole study cohort. Furthermore, age (β = −0.048 *p* < 0.001), menopause (β = −0.747; *p* < 0.001), production history (β = −0.107; *p* = 0.001), a history of hypertension (β = −0.044; *p* = 0.028), and eGFR (β = −0.005; *p* < 0.001), were negatively associated with baseline *T*-score in women. A history of DM (β = 0.124; *p* < 0.001), exercise habit (β = 0.209; *p* < 0.001) and BMI (β = 0.057; *p* < 0.001) were positively related to baseline *T*-score in women. For men, living alone was not significantly associated with baseline *T*-score in multivariable linear regression model. Age (β = −0.038; *p* < 0.001), smoking (β = −0.159; p <0.001), a history of hypertension (β = −0.134; *p* < 0.001), UA (β = −0.012; *p* = 0.025) and eGFR (β = −0.006; *p* < 0.001) were negatively associated with baseline *T*-score in men. Exercise habit (β = 0.329; *p* < 0.001) and BMI (β = 0.032; *p* < 0.001) were positively related to baseline *T*-score in men.

### Correlation between living status and calcaneus ultrasound Δ*T*-score in the whole study cohort

Determinants of the Δ*T*-score in the participants with a median 4 years of follow-up data (*n* = 26,850) were analyzed using multivariable linear regression analysis (Table 4). After adjusting for living status, age, sex, smoking status, exercise habit, DM, hypertension, BMI, fasting glucose, TG, total cholesterol, eGFR, and UA, living alone (β = −0.049; 95% CI = −0.098 to −0.001; *p* = 0.048) was significantly associated with a low Δ*T*-score. In addition, Exercise habit (β = −0.052; *p* < 0.001) and cholesterol level (β = −0.002; *p* < 0.001) were negatively associated with Δ*T*-score. Age ≥60 years (β = 0.031; *p* = 0.042), male sex (β = 0.142; *p* < 0.001), BMI (β = 0.007; *p* < 0.001), and eGFR (β = 0.031; *p* < 0.001) were positively associated with Δ*T*-score.

**Table 4:** Association of living condition and calcaneus ultrasound Δ*T*-score in study participants (*n* = 26,850) using multivariable linear regression analysis.

**Variables**	**Total**		**Woman**		**Man**	
	**Multivariable (**Δ***T*****-Score)**					
	**Unstandardized coefficient (95% CI)**	***p*-Value**	**Unstandardized coefficient (95% CI)**	***p*-Value**	**Unstandardized coefficient (95% CI)**	***p*-Value**
Living alone	−0.049 (−0.098, −0.001)	0.048	−0.035 (−0.106, 0.036)	0.332	−0.066 (−0.152, 0.020)	0.131
Age ≥60 years	0.031 (0.001, 0.061)	0.042	0.139 (0.094, 0.183)	<0.001	−0.061 (−0.104, −0.018)	0.005
Male *vs*. female	0.142 (0.108, 0.176)	<0.001	–	–		–
Smoking history	0.004 (−0.029, 0.037)	0.809	0.028 (−0.037, 0.094)	0.396	−0.008 (−0.044, 0.028)	0.652
Exercise habit	−0.052 (−0.077, −0.028)	<0.001	−0.066 (−0.100, −0.031)	<0.001	−0.004 (−0.041, 0.034)	0.851
Diabetes mellitus	−0.023 (−0.085, 0.039)	0.463	−0.048 (−0.137, 0.042)	0.299	0.042 (−0.040, 0.123)	0.314
Hypertension	−0.013 (−0.051, 0.025)	0.497	−0.011 (−0.067, 0.044)	0.689	−0.004 (−0.054, 0.046)	0.883
BMI (per 1 kg/m^2^)	0.007 (0.004, 0.011)	<0.001	0.008 (0.003, 0.013)	0.003	0.006 (0, 0.012)	0.058
Laboratory parameters
Fasting glucose (per 1 mg/dl)	0 (0, 0.001)	0.223	0 (−0.001, 0.001)	0.750	0.001 (0, 0.002)	0.052
Total cholesterol (per 1 mg/dl)	−0.002 (−0.001, 0)	<0.001	−0.001 (−0.001, 0)	0.018	0 (−0.001, 0)	0.185
Triglyceride (per 1 mg/dl)	0.00004 (0, 0)	0.602	0 (0, 0)	0.262	−0.00003 (0, 0)	0.752
Uric acid (per 1 mg/dl)	0.006 (−0.005, 0.018)	0.253	0.013 (−0.004, 0.029)	0.139	0.002 (−0.012, 0.017)	0.740
eGFR (per 1 ml/min/1.73 m^2^)	0.031 (0.001, 0.061)	0.001	0.001 (0.001, 0.002)	<0.001	−0.001 (−0.104, −0.018)	0.045
Menopause	–	–	−0.125 (−0.084, −0.166)	<0.001	–	–
Production history	–	–	0.054 (0.019, 0.090)	0.837	–	–
Breast-feeding experience	–	–	0.139 (0.094, 0.183)	0.003	–	–

BMI, body mass index; eGFR, estimated glomerular filtration rate.

### Correlation between living status and calcaneus ultrasound Δ*T*-score in the men and women

We separately analyzed the determinants of the Δ*T*-score in the participants with a median 4 years of follow-up data in men and women (Table 4). After adjusting the same confounding variables as Table 3, living alone was not significantly associated with Δ*T*-score in neither women nor men. For women, menopause (β = −0.125; *p* < 0.001) and exercise habit (β = −0.066; *p* < 0.001) were negatively associated with Δ*T*-score. Age ≥60 years (β = 0.139; *p* < 0.001), breast-feeding experience (β = 0.139; p <0.001), BMI (β = 0.008; *p* = 0.003), and eGFR (β = 0.001; *p* < 0.001), were positively correlated to Δ*T*-score. For men, age ≥60 years (β = −0.061; *p* = 0.005) and eGFR (β = −0.001; *p* = 0.045) were negatively associated with Δ*T*-score.

## Discussion

In this study, we explored the association between living status and calcaneus ultrasound *T*-score at baseline and follow-up. A total of 118,835 Taiwanese individuals were assessed at baseline, and 26,850 were followed for a median of 4 years. We demonstrated that living alone was correlated to low baseline *T*-score, and these result consistently shown in the women. Moreover, living alone was related to a greater decrease in *T*-score after 4 years of follow-up.

The number of elderly people living alone is rapidly increasing in most developed countries, including Taiwan (17). Previous studies have indicated that living alone may be correlated to a decreased quality of life, worse physical performance, worse mental health, poor social interaction, and less use of preventative care (25–27). In addition, several studies have investigated the relationship between fractures and living status (28–30). A prospective population-based cohort study in Sweden identified that living alone was an independent risk factor related to incident fractures among 30,446 middle-aged persons during a median follow-up period of 20.7 years (29). However, there is little information on the relationship between osteoporosis and living status. In the current study, we indicated that living alone may be linked to low baseline *T*-score and continuous decline in *T*-score. These findings recommend that more attention should be paid to people who live alone, and that interventions should be implemented to prevent osteoporosis and fractures.

Women and men have different onset and development of osteoporosis, which depend on different risk factors (31). In addition, the bone density of the male heel is greater than that of the female, and the bone marrow in the male heel also has relative amounts of fatty acids different from the female ones (31). Men may have biologic advantages, including greater bone size, more adjacent muscle mass, and a steadier rate of bone loss (31). Therefore, we conduct separate statistical analyses for women and men. We found that living alone was associated with low baseline *T*-score in women, but not in men. Women and men have common risk factors for lower baseline *T*-score including old age, no exercise habit, hypertension history, low BMI, and high eGFR. In addition, women with production history, postmenopausal women, and male smokers may have lower baseline *T*-score. However, living alone was not associated with decrease in *T*-score after 4 years of follow-up in neither women nor men. Postmenopausal women and older men may have decrease *T*-score after 4 years of follow-up. These findings suggested that living status may have different impact on *T*-score between men and women and different factors of osteoporosis between men and women that should be acknowledged.

There are several possible explanations for why living alone increases the risk of osteoporosis. First, adequate intake of calcium, vitamin D, and protein is essential to maintain bone health. However, previous studies have reported that people living alone may have a lower frequency of outdoor activities and consequently less exposure to sunlight, as well as unhealthy eating habits with a less diverse diet and less fish (32). Unhealthy eating habits have also been reported to be more common in people living alone with a lower income (25, 26), which may lead to vitamin D and calcium deficiency and subsequently excessive bone resorption (32). Vitamin D has been shown to play an essential role in calcium storage (33) and osteoporosis may occur if the bone resorption rate exceeds the bone formation rate. Second, falls and fractures are common in people living alone due to the lack of care from others. In addition, people living alone may receive less information about a healthy lifestyle and preventative care (25–27). These observations emphasize the need for interventions and healthcare education for people living alone.

In addition to age, genetic factors and nutrition, lifestyle factors such as smoking, drinking alcohol, chewing betel nut and physical activity also influence bone mass (7). Numerous studies have suggested that unhealthy lifestyle habits including smoking and low leisure-time physical activity can increase the risk of fractures (28, 29). We also found that smoking and a lack of exercise were related to low baseline *T*-score. Preventing falls and stopping smoking are first-line strategies to prevent osteoporosis and fractures (34). A prospective observational study followed 1,033 women aged 75 years for 10 years, and found that smoking cessation reduced the risk of vertebral fractures (35). In addition, smokers living with others may be more motivated to stop smoking (36–38) and family involvement has also been shown to be an effective strategy to help smokers quit. In addition, previous studies have shown that regular physical activity can enhance BMD and help to maintain bone mass, muscle mass and body balance to prevent falls (7, 39, 40). Therefore, maintaining a healthy lifestyle and functional capacity is very important to maintain bone health in people living alone.

Improvements in the planning and implementation of long-term care services for people living alone are an increasingly important issue. In response to the aging population, the Ministry of Health and Welfare in Taiwan developed long-term care policies, which include a community-based integrated care system. The system is a three-tiered service network known as “network ABC”, which aims to integrate community services and supportive care to reduce disease burden and connect discharge preparation services with home care and community care (41). For example, grade C care institutions are the most widely available facilities, which provide meals, arrange puzzle games and simple physical activities during the daytime for elderly people living alone and those who are physically or mentally disabled to help prevent cognitive decline, osteoporosis and sarcopenia. In summary, network ABC addresses aspects of nutrition, medical care, transportation, disease prevention, home accessibility, and respite services for family caregivers. These long-term care policies could effectively decrease the risk of low BMD and prevent subsequent complications.

The major novelties and strengths of this research include the inclusion and follow-up of a large number of participants with regards to the association of living status with calcaneus ultrasound *T*-score. This study has several limitations. Firstly, we only evaluated osteoporosis using ultrasound rather than the dual energy X-ray absorptiometry (DXA). Although BMD is typically assessed using the DXA and the use of BMD *T*-scores is recommended when deciding pharmaceutical interventions for osteoporosis, quantitative ultrasound is a relatively inexpensive and convenient method of measuring *T*-score without radiation exposure. Unlike the DXA that uses X-rays (or ionizing radiation), the ultrasound method, being radiation-free, allows easier monitoring of a large population (42, 43). Furthermore, the method for evaluating bone density with US has been validated by several researches that report a strong and significant correlation between the DXA data and the ultrasound data on the calcaneus (42, 43). In addition, the Achilles InSight system has demonstrated the ability to detect osteoporosis based on axial BMD using DXA in Chinese women (44). Secondly, both ultrasound and X-rays are attenuated not only by bone but also by bone marrow fat (45). Therefore, with the same density of trabecular bone, subjects with higher bone marrow fat would have a higher *T*-score. In particular, with nuclear magnetic resonance spectroscopy, a correlation has emerged between an increase in bone marrow fat and a decrease in BMD (46). In this way, osteopenic and osteoporotic patients are underestimated. One way to overcome this drawback is to use nuclear magnetic resonance, which is another radiation-free technique allowing to take into account both the trabecular density and the amount of fat in the bone marrow (47, 48). Thirdly, the history of falls and fractures was not recorded. Besides, we lacked information on food consumption including calcium & vitamin D intake, and diet is one of the major causal factors for osteoporosis. Another limitation is that we lacked information on medication history, and medications such as anticonvulsants, benzodiazepines, glucocorticoids, immunosuppressants and others, may contribute to the development or prevention of osteoporosis. In addition, all participants were Chinese ethnicity. The incidence of osteoporotic fractures varies markedly between different geographic areas due to racial differences in skeletal size (28). Therefore, our findings may not be applicable to other ethnicities. In addition, potential factors related to osteoporosis including occupation, income, educational level, area of residence, and religious belief may need to be considered in further studies.

## Conclusions

In conclusion, we found that living alone may be correlated to low calcaneus ultrasound *T*-score and decrease in *T*-score in this large population-based longitudinal study. Aside from living status, a lack of regular exercise and smoking were also related to lower *T*-score. To prevent osteoporosis and reduce the risk of fractures in Taiwan, it is important to implement long-term community-based care policies for people living alone, including increasing the amount of activity through regular exercise, and promoting tobacco cessation, healthy eating habits, and control of chronic diseases associated with a deterioration in BMD.

## Data availability statement

The raw data supporting the conclusions of this article will be made available by the authors, without undue reservation.

## Ethics statement

The studies involving human participants were reviewed and approved by Kaohsiung Medical University Hospital (KMUHIRB-E(I)-20210058). The patients/participants provided their written informed consent to participate in this study.

## Author contributions

Conceptualization, methodology, validation, formal analysis, writing—review and editing, and supervision: H-JT and S-CC. Software and investigation: J-HG. Resources, project administration, and funding acquisition: S-CC. Data curation: S-CC and J-HG. Writing—original draft preparation: H-JT and T-YL. Visualization: H-JT. All authors have read and agreed to the published version of the manuscript.

## Funding

This work was supported partially by the Research Center for Precision Environmental Medicine, Kaohsiung Medical University, Kaohsiung, Taiwan, from The Featured Areas Research Center Program within the framework of the Higher Education Sprout Project by the Ministry of Education (MOE) in Taiwan and by Kaohsiung Medical University Research Center Grants (KMU-TC111A01 and KMUTC111IFSP01), Kaohsiung Medical University Hospital (KMUH107-7R79), and Kaohsiung Municipal Ta-Tung Hospital (KMTTH-110-R006).

## Conflict of interest

The authors declare that the research was conducted in the absence of any commercial or financial relationships that could be construed as a potential conflict of interest.

## Publisher's note

All claims expressed in this article are solely those of the authors and do not necessarily represent those of their affiliated organizations, or those of the publisher, the editors and the reviewers. Any product that may be evaluated in this article, or claim that may be made by its manufacturer, is not guaranteed or endorsed by the publisher.
